# Community-engaged Research with Rural Latino Adolescents: Design and Implementation Strategies to Study the Social Determinants of Health

**DOI:** 10.5130/ijcre.v11i1.5721

**Published:** 2018

**Authors:** Megan Comfort, Marissa Raymond-Flesch, Colette Auerswald, Linda McGlone, Marisol Chavez, Alexandra Minnis

**Affiliations:** 1 Behavioral and Urban Health Program, RTI International, 351 California Street, Suite 500, San Francisco, CA, USA; 2 Department of Medicine, University of California, San Francisco, 550 16th Street, San Francisco, CA, USA; 3 Division of Adolescent and Young Adult Medicine, University of California, San Francisco, 3333 California Street, Suite 245, San Francisco, CA, USA; 4 School of Public Health, University of California, Berkeley, 570-D University Hall MC #7360, Berkeley, CA, USA; 5 Monterey County Health Department, Public Health Bureau, Monterey County Government Center 1441 Schilling Place, Salinas, CA, USA; 6 Women’s Global Health Imperative, RTI International, 351 California Street, Suite 500, San Francisco, CA, USA

**Keywords:** community engagement, Latino youth, rural environment, social determinants of health, cohort

## Abstract

The health of adolescents, perhaps more than in any other period of their life, is shaped by the social determinants of health (SDH). The constellation of SDH that disadvantages a specific group’s health may also make members of that population unable or unwilling to engage in health research. To build a comprehensive body of knowledge about how SDH operate within a specific social context, researchers must design studies that take into account how various vulnerabilities and oppressions may affect people’s experiences of being recruited, interviewed and retained in a study. In 2014, we initiated a prospective cohort study with Latino youth living in the agricultural area of Salinas, California. We began this study with the understanding that it was imperative to develop methodological strategies that actively addressed potential challenges in ways that were culturally responsive, community engaged and inclusive. In this article, we describe our approach to developing best practices in four key areas: 1) building community partnerships and engagement; 2) consideration of staffing and staff support; 3) engaging youth’s perspectives; and 4) developing culturally appropriate research protocols. In our sample of 599 participants, nearly all youth identify as Latinx (94 per cent), half (49 per cent) have at least one parent employed as a farmworker, 60 per cent reside in crowded housing conditions, and 42 per cent have mothers who did not complete high school. Given these multiple vulnerabilities, we view a robust number of youth expressing interest in study participation, the willingness of their parents to permit their children to be enrolled, and the achievement of an ambitious sample target as evidence that our efforts to undertake best practices in community-engaged and inclusive research were well received.

## Introduction

Groups of people who are systemically marginalised within a society have poorer health outcomes than those who have access to safe living conditions, non-hazardous work, a living wage, and educational, health and social welfare institutions (Wilkinson & Marmot 2003). These types of resources are frequently considered to be ‘social determinants of health’ (SDH), a term that broadly encompasses ‘the conditions in which people are born, grow, develop, live, work, and age’ (Viner et al. 2012). Importantly, the constellation of SDH that disadvantages a specific group’s health may also make its members unable or unwilling to engage in research: residential mobility, fear of arrest, non-comprehension of a national language, or a lack of transportation can all be barriers to optimal health and to research participation (George, Duran & Norris 2014). This has the potential to more deeply entrench health inequities as populations that go unstudied cannot benefit from interventions, treatments and services tailored to their needs (Dodgson & Struthers 2005; Wilson & Neville 2009).

To build a comprehensive body of knowledge about how SDH operate within a specific social context, researchers must begin by considering how various vulnerabilities and oppressions may affect people’s experiences of being recruited, interviewed and retained in a study. For example, members of a population that have suffered threats of deportation may be wary of interacting with people from outside their community and thus be challenging to reach through standard recruitment and retention channels (Teedon et al. 2015). Likewise, a longstanding history of exploitation of low-resource communities by researchers who were not members of those communities could be another barrier to people’s willingness to participate (Cacari-Stone et al. 2014; Sudhinaraset et al. 2017)Wallerstein et al. 2014, Sudhinaraset, Ling et al. 2017. Such exploitation by outsiders can have a negative impact that reverberates far beyond the research study itself. For example, the disclosure of the infamous Tuskegee Study of Untreated Syphilis in the Negro Male, conducted by the United States Health Service from 1932 to 1972, has been found to be correlated with ‘increases in medical mistrust and mortality and decreases in both outpatient and inpatient physician interactions for older black men’ (Alsan & Wanamaker 2018). A local population’s sense of distrust or wariness about exploitation can also arise in the context of community-based organisations if they are perceived as being overly accountable to political funding sources or other outside interests (Marwell 2004).

If populations underrepresented in research choose to enrol in a study, research or survey questions developed for mainstream populations may not resonate with them or adequately allow for their experiences, which may lead to discomfort, frustration or distress, as well as a perception among community members that research is unhelpful. A lack of consonance between a population and the questions being asked also could result in incomplete or poor quality data, and potentially an investment of funds in research that yields few results of interest or improvements in population health. Under these circumstances, the risk of further stigmatisation and negative labelling of communities through the research process is all too real, and a problem-based approach can create a pathologising lens for researchers that obfuscates the strengths of communities.

In this article, we discuss the *A Crecer* (‘To Grow’) study, which examines SDH among Latino youth living in an agricultural community. We undertook this study with the knowledge that factors which potentially make these youth vulnerable to poor health outcomes may also pose specific challenges to enrolling them as research participants. We therefore understood that it was imperative from the outset of the study to develop methodological strategies that actively addressed these challenges in ways that were culturally responsive, community engaged and inclusive. As we set ourselves to this task, we interpolated our own identities in order to be reflexive about how members of this community would see us as ‘outsiders’, or different from them, as well as our shared experiences and commonalities. Within this article’s authorship team (hereafter referred to as ‘our team’), which was responsible for the design and leadership of the study, two of us identify as Mexican American and two as children of immigrants. Some of our families struggled to make ends meet during our childhoods, including by working in the agricultural field economy, while others of us had the privilege of financial security. Prior to attending college, a few of us lived in neighbourhoods or attended schools that experienced elevated levels of teen pregnancy and violence. None of us grew up in the community where we conducted our research, although one author has lived and worked there for over 30 years and another was raised in a similar community in California.

In designing our approach, we understood that the youth we sought to enrol in our study and their families would consider us to be outsiders, and therefore our methodological strategies needed to address issues of building trust and rapport. With this in mind, we generated a series of questions about best practices for conducting research with rural Latino youth that shaped our research development and implementation: (1) How can we best develop and strengthen *community relationships* and community engagement in the research? (2) What considerations are important when *staffing our study,* and how can we best support staff to do this work? (3) How can we engage *youth’s perspectives* in this study? (4) How can we develop *culturally appropriate research protocols?*

Below we present the methodological strategies developed in response to these questions and used in *A Crecer* to recruit and retain a longitudinal cohort of 599 youth. We begin with a review of the literature to situate our work methodologically within other efforts to engage marginalised and oppressed populations in health research, as well as conceptually at the junction of adolescent health, SDH and rural Latino youth. Next, we provide an overview of the study purpose and settings to contextualise the community in which we work. We then detail our methodological strategies as they relate to our best practice questions. We conclude by reflecting on our experiences undertaking this research, including the importance of sustaining a focus on community strengths and implications of this process for future studies.

### Methodological approaches to engaging marginalised populations in health research

Research with members of marginalised populations has a long and troubled history that emphasises the importance of thoughtful engagement with such groups. Certain populations may be difficult to recruit and enrol in health studies because of well-founded misgivings about the trustworthiness of researchers, but others may be elusive because ‘public acknowledgement of membership in the population is potentially threatening’ (Heckathorn 1997). This frequently includes people who are at risk of arrest, harassment or violence due to a specific activity (e.g. drug use or sex work) or characteristic (e.g. undocumented immigration status). Under these circumstances, participating in research may take on additional risk either because the topic of the study identifies someone as having a vulnerable status, or because being involved in a study could make someone easier to find or to prosecute if there were a breach of confidentiality.

Multiple methodological approaches have been developed in epidemiologic research to grapple with such challenges. Respondent-driving sampling (RDS), for example, constitutes one strategy that engages participants through their trusted social networks, a design that uses structured incentives and chain-referral methods to reach ‘hidden populations’ (Heckathorn 1997). As its name implies, RDS offers a recruitment strategy, yet does not necessarily address issues of marginalisation or vulnerability at other stages of the research process (e.g. study staffing, research instruments).

Another methodological approach that has been adopted for public health research is community-based participatory research (CBPR). A defining feature of CBPR is the cultivation of inclusive partnerships with shared decision-making and ownership of the research process (Israel et al. 2013). Building trust and respect between researchers and communities is foundational to successful CBPR work, yielding research that addresses locally identified needs, recognises a community’s assets, supports co-learning between diverse partners and is positioned to address health disparities (Minkler & Wallerstein 2008). CBPR is particularly effective in informing the development of interventions that address SDH (de Sayu & Sparks 2017), and CBPR methods have been applied to numerous public health issues, with successful examples found in areas ranging from chemical exposure for nail salon workers (Quach et al. 2013) to hepatitis B vaccination (Ma et al. 2017). For research on adolescents, youth participatory action research (YPAR), is a form of CBPR that aims to engage youth directly (Berg, Coman & Schensul 2009; Langhout & Thomas 2010). Several YPAR publications have focused on the opportunities for, and challenges encountered in, building empowerment and leadership development with youth, as well as defining a feasible and actionable research agenda (Madrigal et al. 2016; Ozer et al. 2013).

In this article, we describe our approach to designing and implementing the *A Crecer* study as guided by best practices in community-engaged research inspired by CBPR. The processes of CBPR have been explicated in detail, and there are manuals guiding the use of CBPR approaches (Minkler & Wallerstein 2008). However, applications of the CBPR framework may vary across stages of the research, and some research teams may not be equipped to fully adopt CBPR methods – which can be intensive – at all phases. We recognise that we did not follow the CBPR framework to the extent necessary to exemplify this methodology in its fullest sense. However, through our awareness of our positionality as outsiders to the community and the concomitant need to build trust and rapport, we understood the benefit of incorporating the principles of respect for community stakeholders and responsiveness to community needs. Despite increased use of community-engaged methods in research, there are few examples in the literature outside of CBPR-specific studies that explore how to establish multi-sectoral partnerships, build trust and engagement among partners, and advance a thoughtful research agenda that integrates the insights of local perspectives with research expertise (Farquhar et al. 2014; McQuiston et al. 2005). Our goal in this article is to elucidate our efforts in these domains. Before providing an overview of our study purpose and setting, we situate our work conceptually within the literature on Latino youth in agricultural communities.

### Adolescent health, social determinants, and rural populations

The health of adolescents is strongly shaped by SDH, including proximal SDH, such as family, education, employment and peers, as well as structural factors such as city ordinances, local criminal justice policies and national legislation. There is global and national recognition that the health of young people has generally been neglected, leading to young adults’ ‘surprisingly poor health’ in the words of the Institute of Medicine (Institute of Medicine and National Research Council 2014; Resnick et al. 2012; United Nations Secretary-General 2015). *The Lancet Commission Report on Adolescent Health and Wellbeing* highlights the importance of addressing ‘inequalities in health and wellbeing in socially and economically marginalized adolescents, including ethnic minorities’, among other groups (Kleinert & Horton 2016). Eliminating health disparities in the United States constitutes a national goal reflected in Healthy People 2020, and addressing SDH is a core strategy for achieving this goal (Satcher 2010).

The ability to improve adolescent health is limited by a lack of technical knowledge and capacity to intervene, including a dearth of evidence regarding the effects of SDH on youth and a shortage of evidence-based interventions and policy. The scarcity of research is most evident among the youth who are most affected by inequities, including ethnic minorities, low-income youth and the children of immigrant parents (Auerswald, Akemi Piatt & Mirzazadeh 2017). Latino youth living in rural areas lie at the intersection of these categories. Most of these youth grow up in immigrant families, whether as immigrants themselves or as children of immigrants (Economic Research Service 2017) and frequently one or more members of these families is involved in low-paid seasonal agricultural labour (Lopez & Velasco 2011).

The existing research on Latino youth has focused primarily on urban Latino populations (Raffaelli, Iturbide & Fernandez 2016), but the few studies that have focused on rural youth in the United States have reported similar or higher levels of risk behaviour, particularly alcohol and other substance use, as well as lower uptake of preventive sexual health services compared to urban youth (Roberts et al. 2017; Vielot et al. 2017; Wang, Becker & Fiellin 2013). These studies indicate a clear need for in-depth longitudinal research focused specifically on rural Latino youth.

While proximal individual-level factors associated with risk behaviours may be similar among rural youth to those found in urban populations, current intervention approaches do not address SDH specific to Latino adolescents living in rural areas, such as the influences of the agriculture-based economy and the dominance of Mexican (im)migration. Understanding these underlying contexts and how they might be contributing to health disparities is recognised as vital to achieving substantive improvements in health outcomes (Bronfenbrenner 1994; Krieger 2012; Marmot 2005). Notably, the Centers for Disease Control and Prevention has identified addressing urban–rural health disparities related to health care access as a national priority (Healthy People 2020).

The relationships of structural and interpersonal factors in rural environments to poor health outcomes for Latino adolescents therefore constitute a largely unexamined but vitally important research area. However, conducting rigorous research with rural youth presents numerous challenges that are common to working with other populations facing health disparities. Our goal in *A Crecer,* from a methodological perspective, was to address these challenges by developing a set of best practices for engaging the local community and being inclusive in our approach to conducting rigorous research that would yield knowledge that was useful and actionable. We turn now to the study purpose and setting, followed by a discussion of our methodological strategies and innovations for developing and strengthening community relationships, staffing the study, engaging youth’s perspectives and developing culturally appropriate research protocols.

### Study purpose, setting and sample

*A Crecer* was designed to examine the multi-level factors that influence Latino adolescents’ wellbeing (including resilience, future orientation and educational engagement), as well as the onset of youth violence and sexual health risks associated with teen child-bearing. The study, which began in 2014, focuses on the transition from middle school to high school, a critical developmental period, thus examining trajectories through middle adolescence. Recruitment began in November 2015 and the full sample was enrolled by March 2017. Data collection is ongoing with a cohort of 599 youth. Study participants complete a questionnaire at baseline and then every six months over the course of two years. All study procedures, including written consent provided by study participants, were approved by the RTI International Institutional Review Board.

*A Crecer* is being conducted in Salinas, California, which offers a vibrant environment for a community-engaged study of the protective factors that influence rural Latino adolescent trajectories. Salinas is a migration destination for agricultural sector employment and is home to multiple generations of immigrants. Eighty-nine per cent of Salinas public middle school students identify as Latino, nearly all of Mexican origin (Salinas Union High School District 2017). Salinas has a strong history of community organising for farmworker rights, engaged families and rich cultural ties. However, alongside these assets, youth experience high rates of entrenched poverty (82 per cent of middle school youth are considered socioeconomically disadvantaged), gang exposure and teen child-bearing (County of Monterey 2017; Salinas Union High School District 2017). Like other regions in California’s Central Valley, this area is experiencing tremendous growth in the Latino population (California Department of Finance 2013; Public Policy Institute of California 2012) and has disproportionately high teen birth rates (California Department of Public Health 2011) alongside high rates of youth violence (National Center for Injury Prevention and Control 2010). *A Crecer* aims to inform prevention approaches that strengthen protective factors and mitigate risk through interventions that attend to SDH in Salinas and similar agricultural communities.

**Figure 1:**
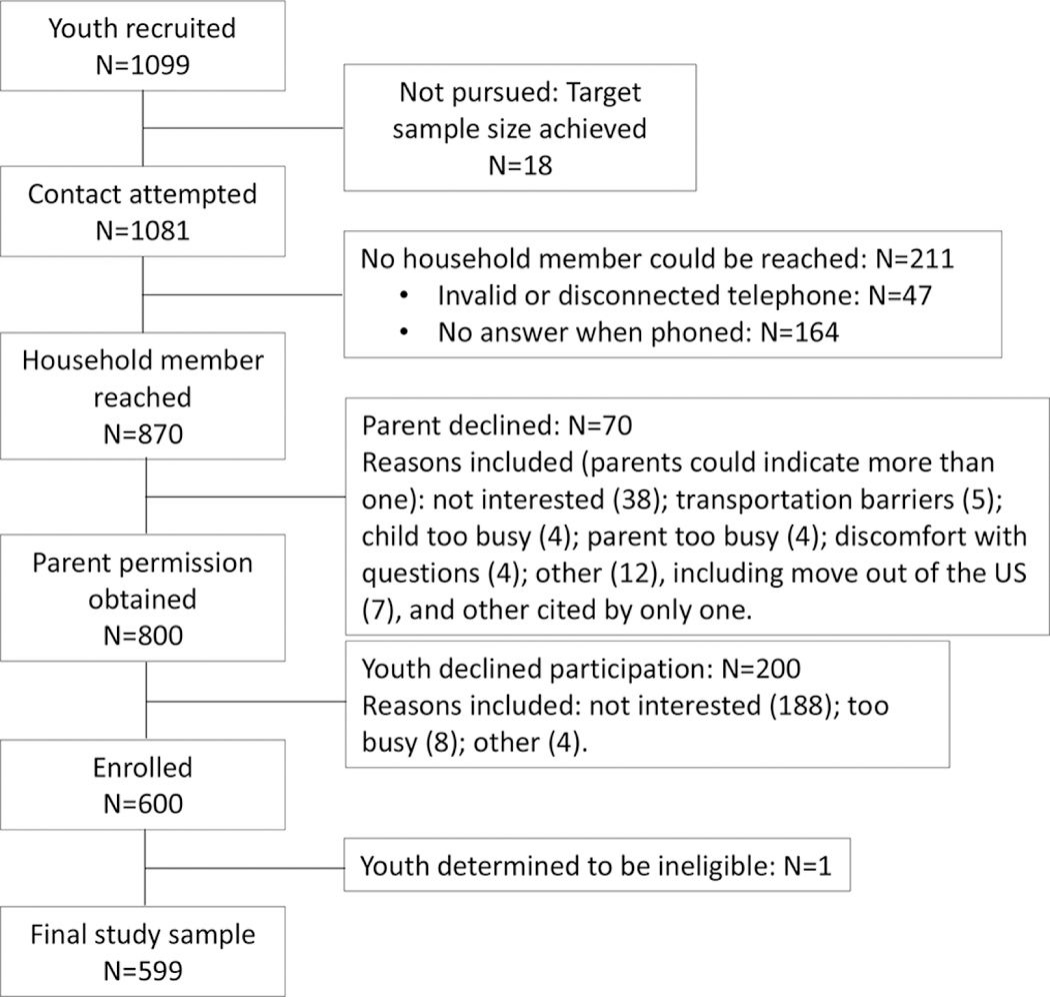
Participant recruitment, household contact, parent permission and enrolment

*A Crecer’s* approach to the cultivation of community partnerships, study staffing, engagement of youth perspectives and development of research protocols contributed to the successful recruitment of our targeted study sample. Figure 1 presents a diagram of enrolment efforts, starting with recruitment of 1099 8th grade youth from the four middle schools that comprise the school district with which we partnered for this study. Youth were approached on school campuses by bilingual research staff members (see ‘Staffing the study’ below) under the leadership of the Project Coordinator (Marisol Chavez). With input from youth advisers engaged during the formative research stage, staff created a brief, interactive ‘pitch’ for the study that they could deliver in classrooms and to small groups of students in schoolyards. During recruitment, eligible youth were asked to provide contact information for a parent who could provide permission for them to enrol in the study. The telephone-based verbal parent permission process was directed by a script that allowed for an interactive conversation with the study staff member initiating the permission process. These conversations were held in English or Spanish, depending on the parent’s preferred language. As depicted in Figure 1, study staff successfully made contact with 870 of the 1081 households. A parent gave permission for their child to participate in 92 per cent of cases. The 600 youth who enrolled in the study did so with an average of 4.9 contact attempts from recruitment through enrolment (median=4; range=1–29). As presented in Table 1, the staff recruited a sample of youth that met the study objectives of balance between male and female youth, geographic distribution of residence within the community and variation in sociodemographic indicators. Nearly all youth identified as Latinx (94 per cent), mostly of Mexican origin. Half (49 per cent) of participants had at least one parent employed as a farmworker, 60 per cent resided in crowded housing conditions (based on the US Census definition) and 42 per cent had mothers who did not complete high school.

Given these multiple vulnerabilities, we see the robust number of youth interested in study participation, the willingness of their parents to permit children to be enrolled and the achievement of an ambitious sample target as evidence that our efforts to undertake best practices for community-engaged and inclusive research were well received. We turn now to describing those efforts.

### Conducting research with rural Latino youth: A Crecer’s approach

As noted, from the outset of *A Crecer* we challenged ourselves to respond to a series of questions about best practices for conducting community-engaged and culturally inclusive research with Latino youth living in Salinas. Understanding our status as people who were not members of this community, we firmly believed in the importance of undertaking research that respected community norms, listened to community voices and addressed questions of community concern. In the following sections, we detail the methodological strategies and innovations we generated and implemented in our efforts to build trust and rapport throughout the design of the study, development of the research protocols and recruitment of the study sample. We begin with our approach to the first question: *How can we best develop and strengthen community relationships and community engagement in the research?*

#### COMMUNITY RELATIONSHIPS AND ENGAGEMENT: DEVELOPMENT OF THE PROPOSAL

The *A Crecer* study developed as a partnership between the Principal Investigator (PI, Alexandra Minnis), who is based in San Francisco (roughly 145 kilometres north of Salinas), and the Monterey County Health Department’s Youth Violence Prevention Coordinator (YVPC, Linda McGlone), who has coordinated teen pregnancy prevention, youth violence prevention and other prevention efforts in Salinas for over 20 years. Through her work, the YVPC has established relationships with diverse leaders and agencies working to improve adolescent health in the community. In 2011, she learned of the PI’s research with Latino youth in San Francisco and invited her to present at the Natividad Medical Center Grand Rounds. This initial meeting led to interest in developing a collaborative research proposal that could examine the intersection of sexual health and youth violence in Salinas. Over the subsequent 18 months, the PI and YVPC established regular professional interactions, exchanging research articles, discussing the community environment in Salinas and planning the grant proposal. As a long-time and well-known Health Department employee with extensive professional network ties in the community, the YVPC was able to actively facilitate early support for the project locally and build connections between the PI and a diverse range of local leaders, including identifying a local co-investigator (Co-I) for the study at Natividad Medical Center.

To inform the research design and refine the research questions in the grant proposal, the PI and YVPC met to discuss potential study objectives and the types of community perspectives that could provide ground-level insights into the core issues and concerns affecting youth in Salinas. In order to learn about those perspectives, they decided to conduct a set of informational interviews with community stakeholders. Drawing on the YVPC’s decades of work in Salinas and strong relationships with community leaders and organisations, they identified key individuals and groups, including directors of youth leadership and gang prevention programs, a middle school teacher, former gang members working to stem gang involvement, a local city council leader, community health clinicians and social service providers. When conducting the initial interviews, they adopted an iterative strategy of asking the key informants to whom they spoke whom else they should approach to discuss the study. In total, they conducted 12 informational interviews. Some of these individuals joined the study’s Community Advisory Board (CAB) and many agreed to support *A Crecer* as partner organisations to facilitate parent engagement and local visibility. The PI and YVPC also solicited youth input through a discussion with peer leaders at a local high school. In addition to these joint meetings, the YVPC helped to mobilise support with the school district, the mayor, the local congressman and the Community Alliance for Safety and Peace, a coalition comprised of over 50 agencies working to reduce violence in Salinas.

This process was crucial to the design of the study as the research questions and approach reflected ideas and reactions offered by the individuals interviewed. Most importantly, the research concept resonated with community stakeholders and stimulated great interest in supporting an effort to generate high-quality data to guide future prevention policies and programs. Many stakeholders discussed community-level influences on youth’s trajectories, including gender and family norms; housing instability and employment structures for farmworker families; intergenerational gang involvement; and differences in access to community resources by immigrant generations. These factors informed our approach to identifying protective and risk factors in youth’s social and structural environments. The *A Crecer* grant proposal was first submitted to the National Institutes of Health in 2012, resubmitted in 2013, and funding commenced in August 2014.

#### COMMUNITY RELATIONSHIPS AND ENGAGEMENT: SCHOOL DISTRICTS AND COMMUNITY STAKEHOLDERS

Once A Crecer funding began, the PI and YVPC focused on strengthening partnerships with the school district and community stakeholders. They jointly attended multiple meetings with school district administrators, during which they presented the project, emphasising how the study goals were aligned with those of the school district for supporting student success. They also met with individual school site teams, including principals, counsellors and parent liaisons (who were responsible for parent–school coordination).

The process of soliciting input from a diverse set of community leaders at the proposal design stage established foundational relationships that supported the development of *A Crecer*’s CAB. Convened by the YVPC and the Co-I, the CAB has met quarterly since the inception of the study. When recruiting members for the CAB, the YVPC and Co-I contacted a variety of colleagues working in youth development, teen pregnancy and violence prevention who were well respected within the community and known to be credible and trusted among local groups. Tapping their networks accelerated development of the CAB, attracting engaged and enthusiastic members. Current CAB membership mirrors the racial and ethnic demographics of Salinas and includes parent representatives, school representatives, a grassroots organiser of women working in agriculture, Salinas service providers, the local hospital’s Co-Director of Community Medicine, and teen radio coordinator of the local bilingual radio station, among others. The CAB advises the study on outreach strategies to parents, as well as recruitment and retention approaches. For example, CAB members suggested the development of a website featuring the logos of member organisations in order to strengthen the perceived legitimacy of the study and deepen community connections; subsequent feedback from participants’ parents indicated that these endorsements boosted *A Crecer*’s credibility as a study that was genuinely invested in the local community. In addition, *A Crecer* data are presented to the CAB for input on interpretation and analysis, which is a particularly important ‘validity check’ for the members of our team who are not based in Salinas. To achieve language inclusivity at the meetings, simultaneous translation in Spanish is offered.

#### STAFFING THE STUDY: COMMUNITY-ENGAGED PROFESSIONAL DEVELOPMENT

In addition to valuing the importance of grounding research in community partnerships, we asked ourselves : *What considerations are important when staffing our study, and how can we best support staff to do this work?* A focal point of our approach to this question has been our commitment to employing young adults from the local community as research staff, particularly because none of the senior staff members grew up in Salinas and we felt it was important to include local voices in our daily work. By bringing community members to the table as salaried employees, encouraging dialogue and soliciting input, we hoped to increase accountability of the research to the people it would ultimately affect. Furthermore, it was congruent with our ultimate goal, as a study seeking to support resilience and opportunities for youth, to invest research funds in creating jobs that could provide exposure to career paths in public health research to current undergraduates and recent graduates from the community.

To maximise visibility, *A Crecer* job announcements were listed online, posted on local community college and state university job websites, sent to the youth collaborative of Monterey County and disseminated by CAB members. In an effort to reach potential applicants who might not otherwise have considered working in research, job descriptions emphasised the desirability of skills such as knowledge of local culture and ability to establish rapport with youth. During the period from the study launch to enrolment of the full cohort, *A Crecer* employed a total of seven Salinas-based staff members, including five originally from the broader Salinas Valley and two from similar communities in California. Six of the seven members of the staff identified as Latinx, and all were bilingual in Spanish and English. Five staff members had attended a local college or university. For five staff members *A Crecer* was their first job upon completion of an undergraduate or Masters program as well as their first experience working in research.

Extensive training in research methods was provided to the *A Crecer* research staff. In-person sessions facilitated by San Francisco-based staff were valuable not only in terms of building capacity and skills among the newly hired junior members, but also in providing opportunities for the junior members to share knowledge about youth in Salinas with the more senior members, and for team bonding. Trainings included topics such as ethics and adherence to Institutional Review Board protocols; effective recruitment techniques; quantitative interview administration; in-depth qualitative interviewing; adolescent development and health issues; SDH research; and how to provide facilitated referrals for distressed participants. Several presentations of local public health data by Monterey County Health Department staff strengthened knowledge of adolescent health inequities. Trainings were ongoing and responsive to the needs of the junior research staff. For example, when staff members remarked that numerous participants were worried about firearm incidents that had taken place in town, an adolescent health physician from the research team (Marissa Raymond-Flesch) conducted a session on techniques for supporting youth exposed to violence and self-care for staff working with vulnerable youth.

#### INCORPORATING YOUTH’S PERSPECTIVES

The question, *How can we engage youth’s perspectives in this study?,* has been central to *A Crecer* from its inception. As a means of learning about youth’s perspectives early in the study, we convened eight focus groups with youth recruited from participating middle schools as well as from Salinas youth leadership programs. (Findings from these focus groups have been published elsewhere; see Raymond-Flesch et al. 2017) Recruitment for these groups was conducted using strategies developed with input from the Principal or Vice-Principal at each school, as well as the CAB. Information sessions, held on campus at lunch, offered students an opportunity to learn about *A Crecer,* sign up for a focus group and talk informally with members of the research staff.

To secure parental support for the focus groups, we solicited input from local implementers of family-based prevention programs and met directly with parents during regularly scheduled parent meetings at each of the middle schools where the focus group recruitment was to be conducted. At each parent group, the study staff introduced *A Crecer* and its objectives; modelled a focus group discussion with the parents, having them role play the activities that would be conducted with participants during the group; discussed parent permission approaches; solicited input on what parents saw as the greatest needs for youth in the community; and offered an interactive educational session on adolescent health issues at a future date. These presentations were either bilingual or conducted in Spanish. Each presentation was led by one member of the San Francisco-based team or the local Co-I and a bilingual and bicultural field coordinator who was from the Salinas community.

**Figure 2:**
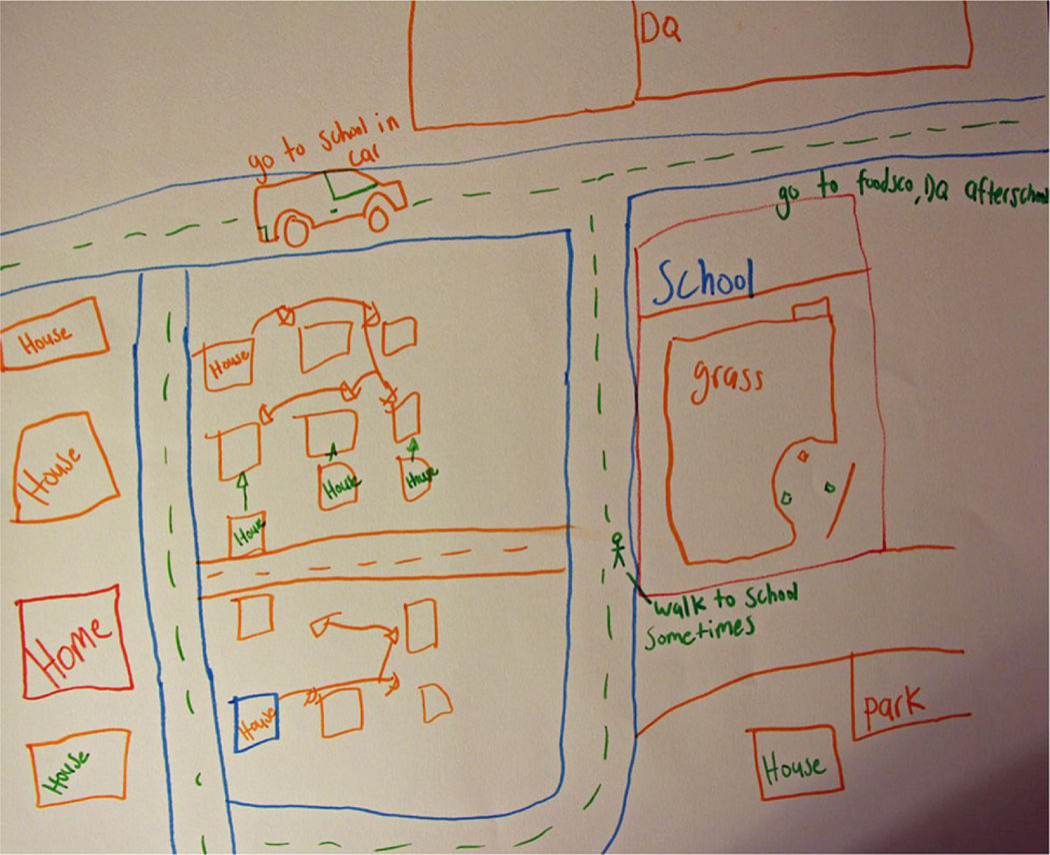
Sample map from focus group

A total of 42 youth participated in the eight focus groups, which followed an innovative structure. Rather than posing questions to the entire group, youth were engaged in a series of activities aimed at generating conversation. In the first activity, participants used stickers to rank their relative agreement or disagreement with statements about family, gender and relationships (e.g. ‘It is very important for a guy to get respect from others’, ‘A woman must be a source of strength for her family’). They were then encouraged to discuss why and how strongly they agreed or disagreed with each statement. In the second activity, participants drew maps of their community, with prompts to include their home, school, recreational areas and transportation methods, and to indicate places where they felt safe and unsafe. Participants then presented their maps to the group, indicating points of interest and providing further details (see Figure 2).

Focus group findings informed questionnaire development and recruitment strategies. For example, youth’s strong orientation towards family as their primary source of health guidance and modelling pathways to adulthood, prompted us to expand the scope of measures related to family included in our questionnaires. In response to requests from parents of focus group participants to have an opportunity to learn more about the research and to contribute to the design of the parent engagement and permission procedures for the cohort study, the Salinas-based Co-I led a meeting for eight parent leaders from two middle schools, two of whom subsequently joined our CAB.

We also convened a Youth Advisory Board (YAB) to provide guidance on study activities. In addition to conducting activities to engage youth in understanding the purpose of the research, the YAB served to inform the development of the study name *(A Crecer)* and logo through an art workshop, the design of study flyers, the development of recruitment messages that would resonate with youth, and the development of the referral guide for youth services and activities in Salinas that is distributed to all participants during their study visits.

#### DEVELOPMENT OF CULTURALLY APPROPRIATE RESEARCH PROTOCOLS

Our final best practices question, *How can we develop culturally appropriate research protocols*?, is of utmost importance to our team. We aim to conduct rigorous research that will yield knowledge that is useful and actionable to those directly concerned with the health and wellbeing of youth in Salinas and similar agricultural areas. It is therefore tremendously important that youth and their parents feel comfortable with, and preferably enthusiastic about, participation in the study. To this end, the *A Crecer* team actively solicited and incorporated information regarding local culture, terminology and norms to inform recruitment and interviewing procedures for the cohort study. We drew heavily on the knowledge and insights of the Salinas-based staff members, led by the Project Coordinator, particularly regarding how to best approach youth and their parents about study participation. Staff framed the study as exploring ‘what it is like to be a teen in Salinas’, and encouraged students to consider participating as a way to ‘share your voice’ and contribute to the community. The staff also noticed that peer leaders could quickly set the tone for whether youth would express interest in study participation, and they consciously adopted strategies of ‘matching the tone’ of exuberant students, generating a sense of shared enthusiasm, while also telling students that they could join the study with friends and come to interview appointments together. As recruitment proceeded, the staff noted that they had developed ways of adjusting recruitment to different environments (schoolyards vs classrooms), groups (‘popular’ kids as compared to quieter youth who kept to themselves) and even schools (noting that students in some schools wanted many details about participation, whereas in others the main focus was on concerns about confidentiality).

Early on in their recruiting, the Salinas-based staff discerned that both youth and their parents had concerns about what was meant by a ‘research interview’. Realising that many people think of an interview in terms of what is seen on television news or talk shows, the staff began proactively explaining the quantitative interview more concretely. For example, they described the survey as ‘multiple choice’ and reiterated that youth could skip any questions they did not want to answer. They also specified that the answers were entered directly into a computer, which youth and parents alike found reassuring, often telling staff that they had been concerned that youth would be asked to write down responses. These explanations also helped parents understand *A Crecer* as a research study, as opposed to an after-school program. In addition, the staff learned that parents were sometimes hesitant to enrol their children in *A Crecer* because they feared negative consequences for their child if they were unable to bring him or her to an interview appointment. The staff therefore made sure to explain to parents that children were never penalised for missing appointments. They also clarified that parents did not need to stay on site during the interview, recognising that many parents had multiple responsibilities and could not spare the time to wait. These efforts to assuage parents’ concerns yielded not only higher numbers of enrolled study participants, but also a sense of pride among students and their parents who began to see study participation as a way of contributing to the community and benefiting youth. Building on this perspective, we partnered with local school officials to arrange for youth to receive community service hours for their participation in *A Crecer*.

Focus group findings also informed procedures for recruitment and obtaining parent permission for the cohort study. Based on parent feedback obtained through recruitment for the focus groups, the staff developed a telephone-based verbal parent permission process that ensured parents had opportunities to talk privately by phone and also to meet staff in person, either at our study office or at one of the five community-based interview locations, in advance of having their child enrol in the study. Concerns about literacy were also raised by youth during the focus groups, which confirmed our decision to use audio computer-assisted self-interviewing (ACASI) for the more sensitive questions, and staff emphasised during recruitment that youth were not required to read or write on their own in order to participate. Finally, we made use of multiple opportunities to build legitimacy within the community. For example, on two occasions, Salinas-based staff members were interviewed by a teen-led youth radio show that aired on a bilingual radio station as a means of raising awareness about the study and demonstrating engagement with the community.

## Discussion

When conducting studies on SDH in communities that experience inequalities and marginalisation, researchers must be mindful of how the same conditions that may shape health disparities may also affect whether and how community members engage with research. We take *A Crecer*’s ability to engage parents and school partners, achieve enrolment targets and sustain high retention to date as indicators that our approach has been successful, not only by research but also by community standards.

Importantly, this engagement has also helped us to ensure that our research focus is well aligned with parents’ and youth’s priorities and community objectives regarding the promotion of adolescent wellbeing. While *A Crecer* brings attention to challenges faced by youth in Salinas and an evidence base for tailored solutions to address these challenges, the study team has been cognisant of the potential for reinforcing a negative image of Salinas through the study findings. In focusing research on two of the most pressing public health problems facing Salinas teens (teen pregnancy and risk of violence), we have strived to also acknowledge the importance of understanding resilience among adolescents who are engaged in school, volunteer in a community to which they feel attached and are connected to families with whom they share strong bonds. It has been a priority of the *A Crecer* team to be vigilant about slipping into a problem-based characterisation of ‘at-risk’ youth in a disadvantaged community, and instead choosing to align with a growing movement to investigate what helps a community thrive, building on the strengths of its residents and a proud cultural heritage. Collaboratively, the research team has challenged itself to incorporate protective factors such as school connectedness, resilience and a future orientation towards the measures of influences that lead to a positive trajectory. This focus grew naturally out of the strong community-research linkages forged by intentional community engagement during the formative stages of the study. This more balanced approach respects and nurtures growing community pride and has been welcomed by CAB members, YAB members and local stakeholders.

Our team is fully committed to the approach described in this article. We recognise that such engagement requires time, resources and a willingness of all parties to listen, be transparent and remain open-minded. It has been important-and meaningful-in *A Crecer* for the San Francisco-based researchers to spend entire days in Salinas in an effort to better understand the context in which the study takes place, meet face to face with community partners and problem-solve on site with the local staff. Likewise, the Salinas-based research staff have worked evenings and weekends in order to maximise their ability to connect with parents, teachers and other community members, shared their observations and experiences in order to inform the aspects of the research that can be adapted to the local culture, and maintained the research protocols that need to be standardised with equanimity and good humour. Within a study that focuses on rural Latino youth, many of whom contend with immigration, acculturation, poverty and other issues that could potentially affect health outcomes, such efforts to create community-level trust and buy-in are key conditions for the production of high-quality data that can be used to support resources, solutions and paths forward.

## Conclusion

Work that will meaningfully affect SDH must be conducted in ways that not only acknowledge community challenges but also recognise and build upon resilience. Taking this approach promotes a growing community awareness of the barriers resulting from SDH and the drive to address these factors to further the development of a safe and thriving community. In the case of our research with rural Latino youth, building in the active participation of the actors in charge of the settings where adolescents live, such as educators and parents, was crucial. Indeed, developing these community partnerships and working to maintain them through accountability and transparency helped expand the reach of our study to a greater number of residents, thereby promoting community engagement and expediting participant recruitment. Likewise, we found that by engaging local opinion leaders – including youth – early in the research process, support for the study spread through multiple channels, minimising the barriers that could have arisen for community members who feel wary of outsiders and instead prompting parents to come forward and enrol their children in what was perceived as a positive community-focused activity.

Despite their importance, building community partnerships and providing avenues to hear the voices of a study population is not sufficient. In order for research among vulnerable groups to be as relevant and ethical as possible, community perspectives and expertise must be deeply integrated into the study itself. Two channels for this are research staff and protocols, the heart and soul of a study. By hiring young people from the local community and valuing and building their knowledge, we hope not only to improve the quality of our current work, but also to help train the next generation of scholars. Similarly, by designing protocols that are culturally appropriate and respectful, we aim to broaden the community’s understanding of research, degree of comfort engaging with it, and expectations that it should lead to solutions that will be feasible and effective. In working towards these goals, we hope that *A Crecer* will live up to its namesake by providing opportunities not only to learn, but for all of us involved – youth, community and researchers – to grow.

## Figures and Tables

**Table 1:** Descriptive characteristics of participants, *A Crecer:* Salinas Teen Health Study

	N	%
Total	599	(100)
Mean age, years (SD)	13.2	(1)
Female	316	(53)
Immigrant generation		
1st: not born in US	71	(12)
2nd: born in US and at least one parent born outside US	427	(71)
3rd+: born in US and both parents born in US	96	(16)
Unknown	5	(1)
Latinx^1^	566	(94)
Mexican origin	531	(89)
Central American origin	64	(11)
Unknown origin	20	(3)
Years lived in US		
Entire life	525	(88)
More than 5 years	55	(9)
5 years or less	19	(3)
Mother’s education		
Less than high school	255	(42)
High school/GED	177	(30)
More than high school	149	(25)
Unknown	18	(3)
Receipt of government assistance past 6 months	320	(53)
Food insecurity (hunger) past 6 months	46	(8)
Household structure		
Contact with father	454	(76)
Lives with father (at least part-time)	545	(91)
At least one parent in agriculture	292	(49)
At least one parent moves for work	87	(15)
Crowded housing conditions	364	(61)

1 Percentages add to ⟶100% as participants may have more than one origin.
